# 

**Published:** 1872-10

**Authors:** 


### ATLANTA
### MEDICAL AND SURGICAL
### JOURNAL.

S.G.O. INDEXED

EDITED BY

JOSEPH P. LOGAN, M.D.,

AND

W. F. WESTMORELAND, M.D.,

*Professor of the Principles and Practice of Surgery in Atlanta Medical College.*

Vol. X.—OCTOBER, 1872.—No. 7.

*PAX ET SCIENTIA, SED VERITAS SINE TIMORE.*

ATLANTA, GEORGIA:

Plantation Publishing Company's Press.

1872.

